# Inosine in Biology and Disease

**DOI:** 10.3390/genes12040600

**Published:** 2021-04-19

**Authors:** Sundaramoorthy Srinivasan, Adrian Gabriel Torres, Lluís Ribas de Pouplana

**Affiliations:** 1 Institute for Research in Biomedicine, Barcelona Institute of Science and Technology, 08028 Barcelona, Catalonia, Spain; sundar.srini@irbbarcelona.org (S.S.); adriangabriel.torres@irbbarcelona.org (A.G.T.); 2 Catalan Institution for Research and Advanced Studies, 08010 Barcelona, Catalonia, Spain

**Keywords:** inosine, deamination, adenosine deaminase acting on RNAs, RNA modification, translation

## Abstract

The nucleoside inosine plays an important role in purine biosynthesis, gene translation, and modulation of the fate of RNAs. The editing of adenosine to inosine is a widespread post-transcriptional modification in transfer RNAs (tRNAs) and messenger RNAs (mRNAs). At the wobble position of tRNA anticodons, inosine profoundly modifies codon recognition, while in mRNA, inosines can modify the sequence of the translated polypeptide or modulate the stability, localization, and splicing of transcripts. Inosine is also found in non-coding and exogenous RNAs, where it plays key structural and functional roles. In addition, molecular inosine is an important secondary metabolite in purine metabolism that also acts as a molecular messenger in cell signaling pathways. Here, we review the functional roles of inosine in biology and their connections to human health.

## 1. Introduction

**Figure 1:**
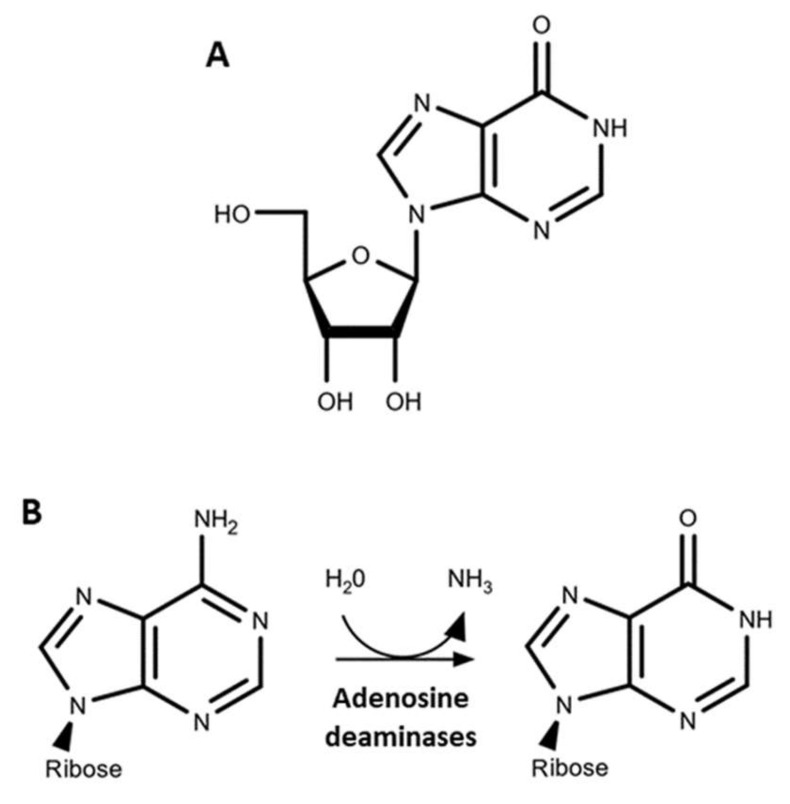
Molecular inosine. (**A**) The N9 nitrogen of hypoxanthine is linked to the C1 carbon of ribose to form inosine. (**B**) Adenosine deaminases hydrolyze the amino group at C6 of adenosine to form inosine.

Inosine was one of the first nucleobase modifications discovered in nucleic acids, having been identified in 1965 as a component of the first sequenced transfer RNA (tRNA), tRNA^Ala^ [1]. Inosine is a purine nucleoside formed by hypoxanthine (IUPAC name: 1, 7 dihydropurin-6-one; molecular formula: C_5_H_4_N_4_O) linked by its N9 nitrogen to the C1 carbon of ribose (Figure 1A).

It has been proposed that life on earth developed either on submarine vents in deep oceans [2] or in warm little ponds [3], and around 3.7 billion years ago [4]. A pre-existent environment containing N_2_, CO_2_, SO_2_, H_2_O, and traces of H_2_ and CO [5] possibly served as a source for the chemical synthesis of nucleobases. Experimental UV radiation of icy mixtures of these molecules may have formed compounds such as 4(3H)-pyrimidone (a precursor of uracil), 4-aminopyrimidine (a precursor of cytosine), and 4-pyrimidinemethanol [6].

The RNA world hypothesis [7] posits that protocells relied on the physico-chemical properties of RNA for catalysis, replication, and selective evolution [8]. However, the actual base composition of RNAs in the RNA world is unknown. Beyond the four major nucleosides (adenosine (A), uridine (U), guanosine (G), and cytosine (C)), extant RNAs typically contain a significant number of noncanonical nucleosides like inosine [9,10], which may have been important for the control of primordial ribozyme activities. Recent discoveries regarding the ability of inosine to improve the fidelity and efficiency of non-enzymatic RNA replication [11] support the possibility that inosine may have been an important component of early nucleic acids [12,13].

Extant non-canonical bases are generated post-transcriptionally by modification enzymes—a process referred to as RNA editing—and play structural and functional roles that depend on both the nature and the position of the modified base. Deamination of adenosines by specific RNA deaminases is the major biological mechanism for inosine generation, through a reaction that converts the 6-aminopurine ring of adenosine to a 6-oxopurine ring (Figure 1B). In extant organisms, molecular inosine serves as a key intermediate in purine metabolism and is a widespread component of various nucleic materials.

In RNAs, inosine plays two major functional roles. Inosine at the wobble position (I_34_) of tRNAs allows the translation of C-, A-, and U-ended codons. This expands the repertoire of triplets that the modified tRNA can recognize and, in doing so, profoundly modifies the balance between codon usage and tRNA abundance in the organisms where the modification is abundant.

In mRNAs, on the other hand, inosine changes the informational content of transcripts, and it can modify the three-dimensional structure of double-stranded regions, thus influencing interactions with RNA-binding proteins. Inosine is interpreted as guanosine by the splicing and translation machineries, affecting transcript localization, splicing, and translation accuracy. Its combined effect upon mRNA and tRNA functions makes inosine a major modulator of translational efficiency and accuracy that contributes to proteome diversity among species.

Here we review the distribution of inosine among extant organisms, and its known biological functions, including its role as an additional regulatory layer for translation, and the links of these functions to human disease.

## 2. Detection and Quantification of Inosine

Since the discovery of inosine by means of laborious purifications of specific RNA species, followed by selective RNA degradation and chromatographic studies [1], a number of techniques now exist for mapping inosine modifications. All these strategies have their strengths and limitations, and their preferential use is dependent on the RNA species of interest and the biological/biochemical question that needs to be addressed. Molecular inosine can be readily detected and quantified using standard biochemical methods that mostly rely on conversion of inosine into hypoxanthine. Detection of inosine within RNA species, on the other hand, is more challenging and will be the focus of this section.

### 2.1. Chromatography-Based Methods

Chromatography is still used today to detect and quantify inosines. It is frequently used when working with in vitro-derived samples (e.g., synthetic or in vitro-transcribed RNAs bearing inosine modifications). The RNA of interest is usually radiolabeled, digested to single nucleotides, and resolved by thin-layer chromatography [14]. This is a semi-quantitative and cost-effective method but cannot be used in a high-throughput manner and does not give information on the location of the modified residue.

To study inosine modifications in in vivo-derived samples (e.g., inosine-containing RNAs derived from cellular extracts), liquid chromatography coupled with mass spectrometry (LC-MS/MS) can be used [15]. This is a highly quantitative non-radioactive method, but it is also low throughput, requires previous purification (in large amounts) of the RNA species of interest, does not give positional information about the modification, and necessitates expensive specialized equipment.

### 2.2. Reverse Transcription (RT)-Based Methods

Several methods for inosine detection and quantification are based on reverse transcription (RT) of RNAs and PCR amplification. Inosine is structurally a guanosine analogue (Figure 1A) that reverse transcriptases read as G instead of the A that it derives from. This artifact can be exploited to detect and quantify inosine by calculating the A-to-G mismatch proportion within PCR products (amplicons), while determining the position of the modifications. A simple, fast, semi-quantitative, and cost-effective method to characterize these amplicons is restriction fragment length polymorphism (RFLP), which can be used when the A-to-I(G) conversion creates or abolishes a restriction enzyme recognition site [16,17,18]. This method allows the evaluation of multiple samples at once but is low throughput in terms of the number of A-to-I edited sites that can be studied.

RT-PCR products can also be sequenced. This can be done by standard Sanger sequencing when only inosines at specific sites and on particular RNA species are evaluated [17,18], and it is a semi-quantitative and inexpensive approach. Most frequently, however, high-throughput RNA sequencing (RNA-seq) is used instead. This is a powerful and highly quantitative technique that allows the identification of multiple inosine sites in a given sample [19,20,21]. However, the method is expensive and requires a good knowledge of analytical computational tools.

Sequencing errors, or A-to-G genomic mutations, may lead to false-positive inosine assignments. To validate whether an A-to-G mutated site is indeed an A-to-I edited site, inosine chemical erasing (ICE)-Seq has been developed [22]. In this method, total RNA is treated with acrylonitrile prior to RNA-seq. This compound cyanoethylates inosines, and the resulting N1-cyanoethylinosines block RT. By comparing RNA-seq data obtained from the same sample with and without acrylonitrile treatments, inosine sites can be unequivocally detected. This method, however, cannot detect sites with 100% A-to-I editing or multiple inosine modifications located in close range.

### 2.3. Other Methods

Specific RNases can be used to cleave inosine-containing RNAs and resolve the digested RNA by gel electrophoresis. These methods are low throughput and not fully quantitative but are simple, inexpensive, and particularly useful when inosine cannot be readily detected by RT-based methods (e.g., certain tRNA species) [23].

For example, RNase T1 is an enzyme that cleaves both guanosine and inosine. It is possible to treat inosine-containing RNA with glyoxal/borate to protect guanosines (but not inosines) from cleavage by RNase T1. In this manner, only inosine-containing sites will be cleaved and can be readily detected [24,25]. Alternatively, endonuclease V (EndoV) specifically cleaves single-stranded RNA at inosine sites, generating fragments that can be detected by Northern blotting [26,27]. EndoV has also been used to develop splinted ligation-based inosine detection (SL-ID). In this method, RNA is treated with EndoV and the resulting (inosine-containing) cleavage products are captured by specific bridge oligonucleotides and splint-ligated to a radiolabeled ligation oligonucleotide, prior to the analysis of the reaction products by gel electrophoresis and autoradiography [23].

More recently, novel developments on Nanopore technologies are allowing the detection and quantification of inosine on native RNAs by high-throughput sequencing without the need of RT [28].

## 3. Molecular Inosine in Metabolism and Signaling

**Figure 2:**
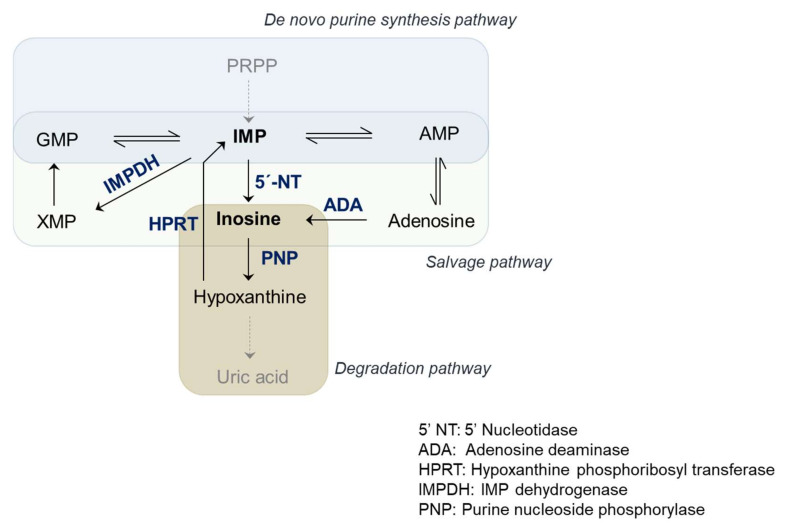
Purine metabolism. Inosine acts as a central intermediate in purine anabolic and catabolic pathways.

Purine nucleotides act as sources of energy, cofactors for metabolic enzymes, and signaling molecules. Accordingly, molecular inosine is a central intermediate in purine biosynthetic and degradation pathways (Figure 2), while also playing an important role in neuronal signaling.

The de novo purine synthetic pathway involves 10 enzymes that sequentially construct purines on the ribose moiety from phosphoribosyl pyrophosphate (PRPP) [29]. Inosine monophosphate (IMP) is the first purine product of this pathway. Highly proliferating cells such as tumor cells adopt an energy-intensive de novo biosynthetic pathway to build IMP. The metabolic enzymes of the de novo synthetic pathway are overexpressed in various cancers [30,31,32,33], and the tumor microenvironment is rich in purine nucleotides [34]. Enzymes involved in folic acid metabolism, such as dihydrofolate reductase (DHFR), play an essential and limiting role in de novo purine biosynthesis. As a result, inhibitors of the de novo purine synthetic pathway, such as antifolates, serve as chemotherapy agents against various cancers [35].

The salvage pathway is a purine anabolic pathway that shares enzymes with the de novo purine synthetic pathway and recycles IMP to replenish the levels of adenosine and guanosine nucleotides. Inosine monophosphate dehydrogenase (IMPDH) and hypoxanthine phosphoribosyltransferase (HPRT) are the key enzymes of the purine salvage pathway. IMPDH converts IMP to xanthine monophosphate (XMP), an immediate precursor to guanosine monophosphate (GMP). The expression of IMPDH is enriched in human leukemic cells and various other cancers [36,37]. Targeting IMPDH is a potential therapeutic strategy for leukemia [38]. Similarly, targeting HPRT with substrate analogs such as 6-mercaptopurine is effective against various cancers and autoimmune diseases [39,40].

In the purine degradation pathway, inosine produced from adenosine is converted by purine nucleoside phosphorylase (PNP) to hypoxanthine, which is further degraded to uric acid [41]. Enhancing the purine degradation pathways is another strategy to reduce the pool of purines of rapidly proliferating cells [42].

Human inosine triphosphatase (ITPase) is a ubiquitously expressed enzyme that hydrolyzes inosine triphosphate (ITP/dITP) to inosine monophosphate (IMP/dIMP) [43]. Functional loss of ITPase can lead to the incorporation of inosines into RNAs and DNAs. ITPase-null mouse embryonic cells show enriched inosine base content in RNAs but not in DNA [44], where it is supposedly removed by DNA repair mechanisms. In humans, recessive ITPase mutations are implicated in pediatric encephalopathies characterized by lack of development, seizures, cardiac abnormalities, and cataracts [45].

In purinergic signaling, nucleotides mediate neurotransmission by serving as signaling molecules to purine and pyrimidine receptor families [46]. Adenosines act as neurotransmitters in both peripheral and central nervous systems [47], and inosine exerts similar effects to adenosine, activating A1, A2A, and A3 adenosine receptors [48]. Inosine administration is neuroprotective in rats with spinal cord injury possibly through its free radical scavenging metabolite, urate [49]. By functioning as an intracellular signaling molecule, inosine also acts as anti-depressant in mice [50], promotes axonal outgrowth, and improves behavioral outcome after stroke [51,52].

Oral administration of inosine has been explored in clinical trials to treat neurological conditions such as Parkinson’s disease (PD). Inosine administration elevates the urate levels in serum and cerebrospinal fluid (CSF), thus affording neuroprotection through radical scavenging [53,54,55]. In PD patients, inosine administration may slow the progression of mental disability, but a phase 3 trial was prematurely terminated as the anticipated efficacy was not met [56]. Inosine pranobex (IP), an inosine derivative, is known for its immunomodulatory and antiviral properties [57] and is being explored for the treatment of COVID-19 in elderly patients for the enhancing effects of IP on lymphocyte proliferation, cytokine production, and natural killer cell cytotoxicity [58].

## 4. Inosine in tRNA

tRNAs are the translators of the genetic code during protein synthesis and are crucial to the efficiency and fidelity of translation [59]. tRNAs fold into a cloverleaf secondary structure and adopt an L-shaped architecture [60] where the nucleobases at positions 34, 35, and 36 form the anticodon that recognizes complementary codon triplets in mRNA. The nucleobases at position 34 do not strictly adhere to Watson–Crick rules when paired with the third base of codons (wobble pairing) in the ribosome.

**Figure 3:**
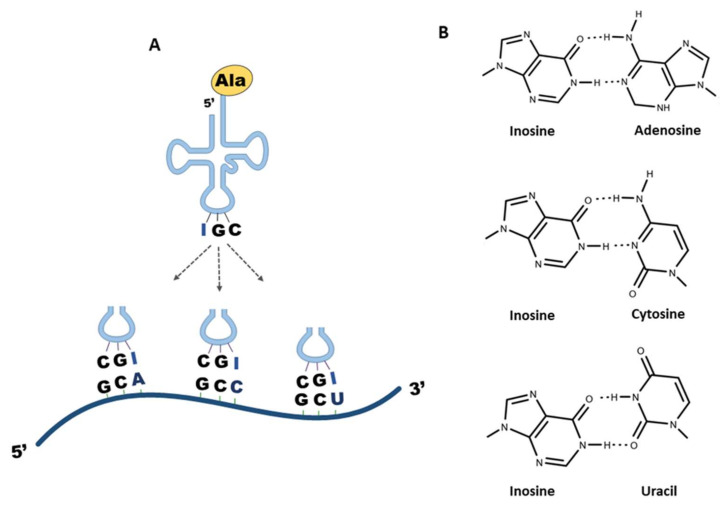
Inosine base pairing. (**A**) Inosine modified tRNA^Ala^ with the anticodon IGC base-pairs with its synonymous codons GCA, GCC, and GCU. (**B**) Hydrogen bonding of inosine with adenosine, cytosine, and uracil.

Adenosines at position 34 (A_34_) of tRNAs are abundant in bacteria and eukaryotes but absent in archaea [61]. A_34_ in tRNAs is almost universally modified to inosine (I_34_) by tRNA-specific deaminases [14,62,63,64,65,66]. I_34_ further potentiates wobble-pairing flexibility of the anticodon, as I_34_-tRNAs recognize A-, C-, or U-ended synonymous codons (Figure 3) [67]. This contrasts with A_34_-tRNAs, which only efficiently recognize U-ended codons.

In a functionally equivalent reaction, uridines at position 34 of tRNAs can be modified by tRNA-specific uridine methyltransferases to form xo^5^U_34_. This modification enables tRNAs to base-pair with A-, G-, or U-ended codons. The nature of the preferred modification at position 34 of tRNAs is a distinguishing feature of archaeal, bacterial, and eukaryotic organisms, and the expansion of I_34_ in eukaryotes was an important influence in the establishment of eukaryotic tRNA gene populations and overall genomic codon usage [68]. Archaea lack both A_34_- and U_34_-tRNA base modifications, whereas extensive U_34_ methylation is a prominent characteristic of bacterial tRNAs [68].

In bacteria, the formation of I_34_ in tRNAs is catalyzed by a homodimeric enzyme: tRNA-specific deaminase (TadA) [14]. Most bacteria have a single A_34_ substrate for TadA: tRNA^Arg^ with the anticodon ACG. However, several bacterial species express more than one A_34-_tRNA. For instance, *Oenococcus oeni* (Firmicutes) contains four A_34_-tRNAs cognate for Arg, Leu, Thr, and Ser. Interestingly, in this species I_34_ has been detected in tRNAs cognate for Arg and Leu but not in A34 tRNAs for Thr and Ser, indicating that the expansion of tRNA substrates modified by tRNA deaminases likely starts with the emergence of unmodified A_34_-tRNA substrates [65]. TadA is an essential enzyme in *Escherichia coli*, a fact attributed to the importance of I_34_-tRNA^Arg^ in translation [14]. In agreement with this, those bacterial species that lack A_34_-tRNA genes also lack TadA [65,69,70,71]. In these species other tRNA isoacceptors compensate for the lack of A_34_-tRNA genes. A second function of TadA-dependent inosine deamination in *E. coli* is discussed later in this manuscript [72].

In eukaryotes, the situation regarding I_34_-tRNAs is more complex than in bacteria, because the eukaryotic adenosine deaminase acting on tRNAs (ADAT) deaminates A_34_ in multiple tRNAs (seven tRNAs in some fungi and plants, and eight tRNAs in most well-characterized species) [73]. Eukaryotic ADAT emergence was accompanied by a dramatic genomic enrichment in A_34_-tRNA genes [68,73,74,75].

Eukaryotic ADATs are heterodimeric enzymes that evolved from the duplication of a bacterial *tadA* gene. The catalytic subunit is known as ADAT2, while its tRNA-binding partner is named ADAT3. A conserved proton-shuttling glutamate, which is essential for the catalytic activity of ADAT2, was lost during the evolution of ADAT3, rendering this subunit catalytically inactive [76,77]. A recent study on the crystal structure of ADAT2/3 from *Saccharomyces cerevisiae* suggests that the positively charged residues at the N-terminal region of ADAT3 may play a role in substrate recognition [78].

It is unclear how ADATs evolved to expand their substrate specificity for multiple tRNAs. In *Trypanosoma brucei*, in addition to A_34_ modification in tRNAs, ADAT2/3 carries out C-to-U editing in single-stranded DNAs [79] and the same enzyme is necessary for the C-to-U editing in tRNA^Thr^ [80]. In this species, substrate recognition by ADAT requires a KR domain containing stretches of Arg and Lys at the C-terminal of ADAT2 [81].

**Figure 4:**
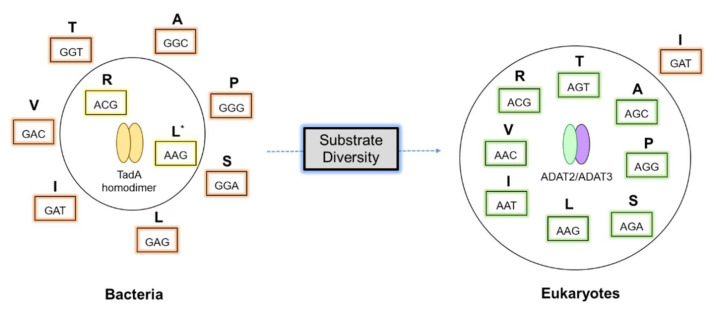
Substrate diversity of tRNA-specific deaminases. In bacteria, tRNA^Arg^_ACG_ is a well-established A_34_ substrate for homodimeric TadA. * Inosine modified tRNA^Leu^_AAG_ is also found in a few prokaryotes such as *O. oeni* [65]. In eukaryotes, diverse A_34_ tRNAs serve as substrates for heterodimeric ADAT2/3 where the population of G_34_ tRNAs is limited. The expansion of A_34_ tRNA diversity co-evolved with multisubstrate specificity in ADATs. (The anticodons are boxed, and the corresponding amino acids are one-letter-abbreviated).

In fungi, reduced levels of I_34_-tRNAs arrest the cell cycle of *Schizosaccharomyces pombe*, and the deletion of the enzyme is lethal in *S. cerevisiae* [82]. Inosine modification in tRNA^Arg^ of *Arabidopsis thaliana* chloroplasts improves the efficiency of translation of the organelle’s genome [83]. The ADAT activity is, in fact, essential in all tested eukaryote species [21,66,79,82], which is to be expected given the fact that most eukaryotic genomes lack genes coding for several G_34_-tRNAs. Therefore, A-to-I editing is required to compensate for the lack of G_34_-tRNAs otherwise needed to decode C-ended codons [74]. In bacteria, on the other hand, G_34_-tRNAs are abundant because prokaryotes have not adopted I_34_ as a general solution for the translation of C-ended codons (Figure 4). Interestingly, G_34_-tRNAs were shown to be toxic to eukaryotic cells as they are prone to induce miscoding in the context of eukaryotic translation systems [84].

Codon composition and RNA structure are important factors that influence translation rates, and the clustering of rare codons (codons that have few copy numbers of cognate tRNAs) in regions of mRNAs limit the rate of translation [85,86]. Thus, the translation of genes rich in ADAT-sensitive codons (codons translated by I_34_-tRNAs: amino acids T, A, P, S, L, I, V, and R, hereinafter TAPSLIVR) might benefit from the increased decoding capacity of inosine-modified tRNAs [64,87,88]. In agreement with this prediction, self-renewing embryonic stem cells that express a large number of genes enriched in ADAT-sensitive codons display enhanced ADAT2 levels [89].

In general, eukaryotic proteomes are highly enriched in protein sequences with ADAT-sensitive amino acid stretches when compared to bacterial proteomes (~4-fold enrichment) [90], and the codon composition of the transcripts coding for TAPSLIVR-rich proteins is biased in favor of I_34_-tRNA dependent codons (~70% enrichment) [90]. Thus, eukaryotes (that preferentially use I_34_-tRNAs for decoding TAPSLIVR, Figure 4) display different proteome composition in terms of proteins rich in TAPSLIVR amino acids as compared to bacterial species, and their transcripts are also enriched in codons that require this modification [65,90,91]. We have proposed that inosine at position 34 of tRNAs represents a eukaryote-specific evolutionary trait selected because it contributes to proteome complexity expansion [92].

Interestingly, I_34_-tRNAs are prone to internal cleavage by endonuclease V, a highly conserved ribonuclease, that cleaves inosine-modified tRNAs at their anticodon [26]. Stress conditions such as oxidation and starvation can trigger the cleavage of tRNAs at their anticodon loops, and the resulted fragments play a number of regulatory roles that are, as of yet, largely unexplored [93,94,95].

**Figure 5:**
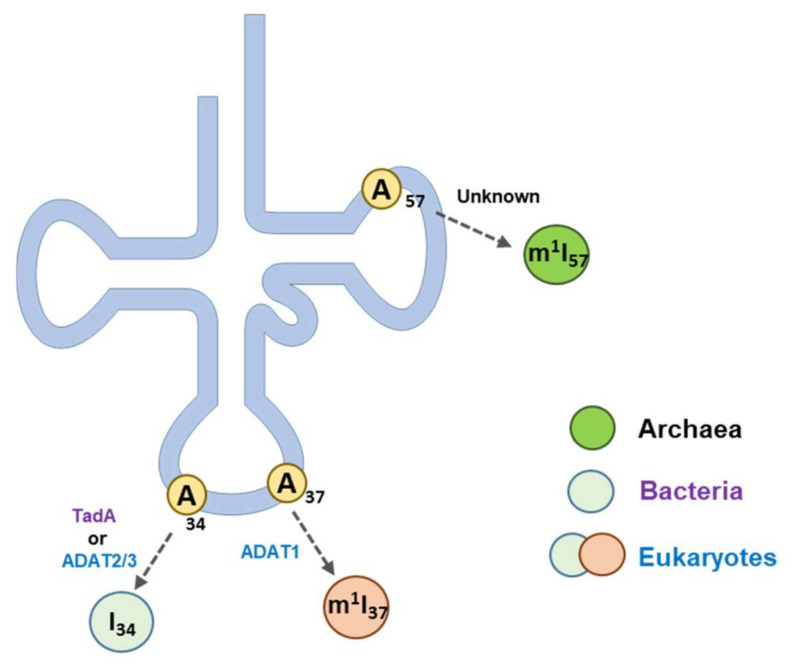
A-to-I editing in tRNAs. Adenosines at positions 34, 37, and 57 of tRNAs can be modified to inosines. The A_34_-to-I_34_ modification is observed in bacteria and eukaryotes and is catalyzed by the enzymes TadA and ADAT2/3, respectively. ADAT1 catalyzes A_37_-to-I_37_ modification in eukaryotic tRNA^Ala^. I_57_ modification was observed in archaeal tRNA^Ile^. Adenosines at positions 37 and 57 are modified to methylated forms of inosine.

In addition to I_34_, adenosines at positions 37 and 57 are modified to methylated forms of inosine (Figure 5) [96]. Methyl-inosine 37 (m^1^I_37_) is only found in eukaryotic tRNA^Ala^ and is formed through a two-step process that requires, first, the tRNA-specific deaminase ADAT1 and, second, the tRNA methyltransferase 5 (Trm5) [97,98]. Methyl-inosine 37 is believed to prevent translational frameshifts and improve translation accuracy [76,97]. Methyl-inosine 57 (m^1^I_57_ or m^1^Im_57_ [99]) was identified in archaeal tRNA^Ile^, and its function is, as of yet, unclear [100].

Mutations in tRNA-modifying enzymes cause serious clinical conditions in humans [63,101], including neuronal degeneration [102]. Point mutations in ADAT3, the partner protein of heterodimeric ADAT, cause intellectual disability and strabismus [103,104,105,106,107], and an 8 bp duplication in the ADAT3 gene has been shown to cause mild intellectual disability [108].

## 5. Inosine in mRNA

Bass and Weintraub first identified A-to-I editing in *Xenopus laevis* double-stranded mRNAs [109]. Since then, more than 36,000 non-repetitive A-to-I editing sites (excluding Alu repeats) have been predicted in the human genome [110].

In eukaryotes, inosines in mRNAs are generated through the activity of adenosine deaminases acting on RNAs (ADARs) [111], which are widely conserved across the eukaryotic kingdom. There are three vertebrate ADAR enzymes (ADAR1, ADAR2, and ADAR3), of which ADAR3 is apparently catalytically inactive [112]. Although ADAR-mediated RNA editing is the main mechanism for inosine introduction in mRNAs, RNA polymerase can occasionally introduce inosines on elongating transcripts [113].

Structurally, inosine alters the stability of double stranded RNA (dsRNA) in a manner that depends on the nucleotide it pairs with. For instance, the I–U base pair is less stable than A–U, whereas the I–C pairs are more stable than A–C pairs [114]. The effects of inosine modification on mRNA structure and function also depend on its position on the mRNA (i.e., untranslated regions (UTRs), introns, and coding regions).

During translation, tRNAs recognize inosines in the coding regions of mRNAs as guanosines. Thus, the modification of adenosine to inosine in mRNA has the potential to generate substitutions in the protein sequence. Inosine editing in a coding region was first reported in the mRNA for subunit 2 of AMPA glutamate receptors (GluR-B) [115]. ADAR-mediated editing of a CAG codon (Gln) to CIG (CGG, Arg) in these transcripts modulates calcium permeability [115]. Similar ADAR-editable sites are present in serotonin receptors [116], squid potassium channel [117], *Xenopus* basic fibroblast growth factor [118], sodium channels in *Drosophila* [119], and various other proteins of physiological importance [120].

In addition to protein recoding, in vitro hypermodification of inosine in the coding region of mRNA may lead to ribosome stalling and truncation of peptides. In particular, an INI codon (codon with inosine as a first and third base) results in the truncation of peptides by 60–80% when compared to the codons with single inosine [121]. In yeast, the inosine-mediated synonymous codon changes do not result in protein recoding but they may affect the RNA stability and the translation efficiency, depending on tRNA availability and codon usage for the modified codon [122], and the same was identified in mouse oocytes [123,124].

A-to-I editing of GluR-B mRNA is required for brain function, and mice with depleted modification levels present with severe seizures and premature death [125,126]. Remarkably, altered A-to-I editing in the pre-mRNA transcripts of serotonin 2C receptors was reported in suicide victims with a history of depression [127].

**Figure 6:**
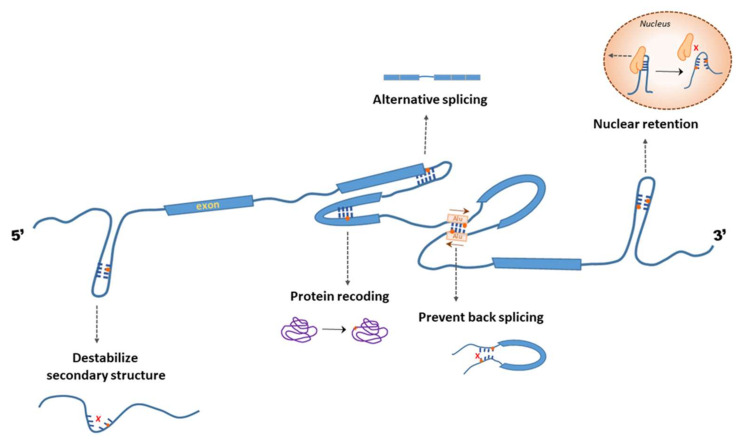
A-to-I editing in mRNAs. The effects of A-to-I editing in different regions of a hypothetical mRNA are shown. Inosine modification in the coding regions may result in protein recoding. Inosines from the untranslated regions modulate the secondary structure of mRNA, thus affecting the localization, stability, and splicing.

In eukaryotic transcriptomes, A-to-I editing is widespread in noncoding regions of transcripts. Inosines in untranslated regions (UTRs) and introns modulate the stability, localization, and integrity of the transcripts (Figure 6) [128]. For example, inosines within the 3′ UTR may result in nuclear retention of the mRNA [129,130]. This was first discovered in mice, where A-to-I editing regulates the nuclear retention of an 8 kb poly(A)+ RNA [129]. However, posterior analyses have reported cytosolic distribution of multiple mRNAs with hyperedited 3′ UTRs in *Caenorhabditis elegans* and *Homo sapiens* [131], indicating that nuclear retention is not always a consequence of 3′ UTR inosines.

A-to-I editing in introns of pre-mRNAs can modulate their splicing because inosines are recognized as guanosines, thus creating or removing alternate splicing sites. The modification of splicing sites by inosine can lead to the translation of alternative reading frames, a phenomenon first observed in mitochondrial transcripts of the *Trypanosoma* mitochondrial *CoxII* gene [132]. In ADAR2 mRNA itself, an intronic inosine modification generates a highly conserved alternate 3′ splicing site that results in the addition of 47 nucleotides to the mature transcript, shifting the reading frame and reducing ADAR2 protein levels [133]. Interestingly, this effect also modulates A-to-I editing levels in pre-mRNA, as higher spliceosome activity limits A-to-I editing by restricting the spatial access of the editing enzymes to the transcript [134].

In *C. elegans*, deletions of the ADAR gene affect vulva development and chemotaxis [135], while ADAR-deleted *Drosophila* mutants exhibit paralysis, uncoordinated locomotion, and tremors caused by the depletion of inosine at 25 sites in transcripts coding for three different ion channels [16]. In mice, ADAR1 inactivation results in an embryonic-lethal phenotype, which is a consequence of the activation of interferon- and dsRNA-sensing pathways [136], liver disintegration [137], aberrant hematopoiesis, and increased apoptosis [138,139].

In humans, the amount of A-to-I editing on mRNAs is strongly tissue-dependent (higher in the brain and thymus, and lower in transformed cells) [140]. The contribution of A-to-I editing to the translation of proteins of oncogenic importance is explored in cancer research for its diagnostic and therapeutic potential [141,142]. For instance, progression of gastric tumor from healthy tissue is associated with enhanced editing at ADAR1-specific sites and downregulation of editing at ADAR2-specific sites [143], pointing at inosine modifiers as potential biomarkers for gastric cancer [144]. Moreover, increases in ADAR1 activity through gene amplification enhance lung tumorigenesis [145], while loss of ADAR1 function allows tumor cells to overcome resistance to immunotherapy by removing the checkpoint that restrains the dsRNA-mediated immune response pathway [146].

Point mutations in ADAR1 are observed in patients with genetic disorders such as Aicardi–Goutières syndrome (AGS), characterized by aberrant immune response mediated encephalopathies [147]. Hyper-mutated ADAR1 is associated with an autosomal dominant condition known as dyschromatosis symmetrica hereditaria (DSH), a phenotype with varied hyper- and hypopigmentation in skin [148].

Though inosine modification in mRNAs is mostly confined to eukaryotes, a recent study identified inosine in the transcripts of *hok*-like genes in prokaryotes. [72]. Interestingly, *E. coli* TadA recognizes a hairpin structure in the coding region of *hok* transcripts (*hokB*, *hokC*, *hokD*, *hokE*) that resembles the anticodon stem-loop of tRNA^Arg^. The editing event in *hokB* recodes a TAC codon (Tyr) to TIC (TGC, Cys), and this *hokB*-Cys29 variant is more toxic to *E. coli* than proteins from unedited transcripts [72]. Levels of *hokB*-Cys29 increase with rising cell density [72] in a mechanism thought to mediate programmed cell death and antibiotic tolerance in bacteria [149,150].

In summary, the regions of mRNAs that form a secondary structure with an editable sequence can be subjected to A-to-I editing by ADARs. Inosine is interpreted as guanosine by the molecular machineries acting on mRNAs. Inosine modifications at the coding regions of mRNA lead to amino acid substitutions in the protein sequence and at the noncoding regions of mRNA modulate the stability, splicing, and transport of mRNAs.

## 6. Inosine in MicroRNAs

MicroRNAs (miRNAs) are short, single-stranded non-coding RNAs that attenuate translation via RNA interference (RNAi). RNAi is the process of posttranscriptional gene silencing through the action of the RNA-induced silencing complex (RISC), which involves the pairing of complementary regions (between position 2 and 8) of miRNAs (known as seed regions) with the target transcripts. Interactions with miRNAs mark mRNAs for translation repression or degradation.

The primary miRNA (pri-miRNAs) transcripts are first cleaved by the ribonuclease Drosha to produce pre-miRNAs, which are further processed by Dicer to generate mature miRNAs [151,152]. Pri- and pre-miRNAs that form secondary hairpin-like structures are targets for editing by ADARs [153]. Interestingly, ADAR1 forms a complex with Dicer that promotes the processing of miRNAs [154]. More than 130 A-to-I editing sites have been identified in miRNAs [155], and these modifications reduce miRNA function by impairing their ability to form RNA duplexes with target mRNAs.

A-to-I editing in miRNAs was first identified in miR-376, a repressor of phosphoribosyl pyrophosphate synthetase 1 (PRPS1) translation. ADAR-mediated editing of miR-376 RNA clusters perturbs its function, and ADAR2-null mice show increased PRPS1 levels [153]. The extent of editing varies with the species and tissue types. Among the miR-376 RNA clusters, 41% of miR-376a1-5p and 92% miR-368-3p are edited in the human medulla oblongata, whereas in mice, 56% of miR-376c-3p and 54% of miR-376a1-5p are edited in the cortex and kidney, respectively [153]. Adenosines from the UAG motifs located in secondary structures of miRNAs serve as targets for ADARs in a tissue-dependent manner [156].

Translation regulation via A-to-I editing of miRNAs has profound effects on tumor progression and metastasis. A-to-I edited miR-200b promotes tumor progression, as the ability of miR-220b to repress ZEB1/ZEB2 transcription factors is modulated [157]. On the other hand, ADAR1-mediated A-to-I editing in miR-376a impairs the translation repression of the glioblastoma tumor suppressor RapA (RAP2A). Strikingly, A-to-I editing enables an isoform of miRNA miR-376a to target autocrine motility factor receptor (AMFR) in glioblastoma cells [158]. AMFR, an internalizing surface receptor, is not a target for unedited miR-376a and its upregulation is correlated with advanced stages of several cancers [159]. Therefore, inosines in the seed regions of miRNAs either attenuate their interaction with target mRNAs or enable them to obtain new target transcripts, with consequences that depend on the function of the target mRNA.

## 7. Inosine in Viral RNAs and Mobile Elements

### 7.1. Inosine in Viral RNAs

Adenosines in viral RNAs can be modified to inosines by hosts’ deaminases upon infection, a process initially identified in samples of human brains infected by the measles virus [160]. Along with A-to-G transitions (a hallmark of inosine modifications), U-to-C conversions were also enriched in the reverse-transcribed cDNA of viral matrix genes from the same samples [160,161]. ADAR1-mediated A-to-I hyperediting weakens the pathogenesis of lymphocytic choriomeningitis virus (LCMV), resulting in nonfunctional viral glycoproteins [162]. In contrast, inosine hypermodification of viral transcripts represses the immune response by masking the transcripts from Mda5, a cytoplasmic sensor that regulates the synthesis of interferons and other inflammatory proteins [136,163].

Inosine also plays an interesting role in viral hepatitis. Co-infection of hepatitis δ virus (HDV) with hepatitis B virus (HBV) increases the risk of severe liver damage in hepatitis patients [164]. The subviral pathogen HDV encodes only one protein, namely hepatitis delta antigen (HDAg), in two isoforms. The shorter isoform (HDAg-S) assists in replication, whereas the longer isoform (HDAg-L) inhibits replication and promotes viral assembly [165,166]. An A-to-I editing event in HDAg transcripts modifies an amber stop codon UAG to UIG (UGG, Trp) by 40% to 60%, leading to an enrichment of the longer isoform HDAg-L [167]. The predominance of HDAg-L diminishes the virulence of the infection, maintaining viral titers constant due to an imbalance between replication and viral assembly.

### 7.2. Inosine in Mobile Elements

Retrotransposons (class I elements or retroposons) are mobile insertion sequences of an approximate length of 300 bases with the capacity to integrate themselves into different parts of the genome via an RNA intermediate. Short interspersed elements (SINEs) are one of three major subclasses of retroposons [168] and constitute up to 11% of the human genome, with over 1 million copies of Alu elements typically found in each genome. These retroposons are derived from 7SL RNA and emerged around 65 million years ago during early primate evolution [169]. Alu elements are abundant in UTRs and intronic regions of mRNA, and their genomic re-integration generates new exons and plays a major role in species evolution [170]. Inosine modifications in intronic Alu elements modify splicing sites and generate new exonic sequences. For instance, A-to-I editing led to the inclusion of a primate-specific Alu-exon in the human nuclear prelamin-A recognition factor by altering a splicing site in its RNA intermediate [171].

A-to-I editing in Alu elements was first discovered in the early 2000s [114]. More than 100 million Alu-RNA editing sites can be detected in human genes [172]. Integration of an Alu element in the opposite direction to another Alu element at a short distance results in the formation of a loop-like structure that serves as a substrate for ADARs [173] and the introduction of inosine at these sites disrupts base-pairing patterns and destabilizes their secondary structure, with gene-dependent effects.

For example, the interaction of two inverted Alu elements flanking an exon may result in the formation of circRNAs by a mechanism called backsplicing (Figure 6). The formation of circRNA structures affects the splicing and nuclear export of transcripts. A-to-I editing suppresses the formation of complete circRNAs and promotes the standard splicing and export of the modified mRNAs [174].

RNA editing at Alu elements embedded in 3′ UTR of dihydrofolate reductase (DHFR) aids the transcript to escape from miRNA-mediated silencing [175]. On the other hand, ADAR interactions with the Alu site may modify the properties of the transcript. For example, the association of ADAR1 with the inverted Alu elements at the 3′ UTRs of proto-oncogenes XIAP2 and MDM2 suppresses their apoptotic inhibitory functions [130].

## 8. Inosine in Other Nucleic Acids

### 8.1. Inosine in Ribosomal RNAs

The presence of inosine in ribosomal RNA (rRNA) is not well reported. In rRNA of *Crithidia fasciculata,* O^2^’-methylinosine was first identified, but the role of this nucleoside in ribosomal structure and function is unclear [176]. Four decades later, transcriptome analyses of *Diplonema papillatum* mitochondria identified inosine in mt-SSU rRNA, where it is proposed to destabilize the structure of rRNA [177], but the function of these inosines and the identity of the enzymes editing the rRNA remain unclear.

### 8.2. Inosine in DNA

Deoxyinosines are observed in DNA, where they are introduced by various independent mechanisms. On the one hand, nitrosative compounds released by macrophages, or exposure to exogenous agents such as nitrous anhydride, can deaminate adenosine to inosine in DNA [178]. Adenosines from DNA strands that form DNA/RNA hybrids are also found to be edited by ADARs [179]. Alternatively, the nuclear accumulation of dITP due to the loss of functional ITPase can lead to the misincorporation of deoxyinosines into newly synthesized DNA [113,180]. These events can lead to point mutations in DNA as deoxyinosine preferentially pairs with cytosine than thymine [181].

DNA repair mechanisms remove deoxyinosine by base excision repair (BER) or alternate excision repair (AER). In BER, alkyl-adenine DNA glycosylase cleaves the N-glycosidic bond between hypoxanthine and the sugar moiety and releases the modified base from DNA. Then, AP lyase seals this apurinic site with adenine using the information from the complementary strand [182]. In AER, endonuclease V creates a nick by hydrolyzing the second phosphodiester bond in the 3′ direction from deoxyinosine. A 3′–5′ exonuclease cleaves the nucleotides at the nicked site [183]. A segmental gap created by this excision is elongated by DNA polymerase with the help of a complementary strand [184].

Interestingly, targeted inosine modifications in DNA have significant implications in gene editing. The bacterial tRNA-specific inosine modifier, TadA, can be synthetically fused to catalytically impaired CRISPR-cas9, which can be programmed to modify selective adenosines to inosines in DNA [185]. Such inosines will be recognized as guanosines by polymerases leading to converting the A·T base pair to G·C. This inosine modification machinery can be exploited in gene therapy to correct disease-causing point mutations.

## 9. Concluding Remarks

Since its discovery in 1965 in yeast tRNA^Ala^, inosine has emerged as a universal and widespread component of nucleic acids with a heterogeneous set of functions and activities. Inosine is present in a range of RNA molecules, where it modulates the efficiency and accuracy of translation, as well as several other biological activities. Moreover, inosine is an important intermediary in purine biosynthetic pathways and a secondary metabolite of purine degradation. Because of its biochemical similarity to adenine, molecular inosine plays a number of physiological roles, such as acting as a neuroprotective purine analog during purinergic signaling.

Inosine modifications in anticodons of tRNAs expand their decoding capacity by their multi-base-pairing chemistry and improve the efficiency of translation. In eukaryotes, tRNAs with inosine at position 34, and cognate for several amino acids, compensate for the absence of tRNA isoacceptors with G_34_ to decode C-ended codons. The relevance of this function is reflected in the neurological disorders caused in humans by mutations in ADAT, the enzyme catalyzing the I_34_ modification.

By mimicking guanosine, and depending on their localization, inosines in mRNAs modulate translation accuracy, splicing, and nuclear export. Defective A-to-I editing in the GluR-B receptor leads to motor neuron death in sporadic amyotrophic lateral sclerosis (ALS), and altered inosine modification levels in the transcripts of serotonin-2C receptors are associated with neuropsychiatric disorders. The physiological importance of inosine, and of the specific proteins whose synthesis is regulated by this modification, turn ADARs (the enzymes responsible for inosine modifications in mRNAs) into promising pharmacological targets.

In miRNAs, A-to-I editing can either impair the ability to repress target translation or enhance their repertoire of target transcripts. The ability of inosine modifications to modulate miRNA function in highly proliferating cells, including silencing oncogenes and tumor suppressors of various cancers, highlights the therapeutic potential of targeting adenosine deaminases as targets for chemotherapy. Indeed, suppression of ADAR activity sensitizes tumor cells and virally infected cells to the immune response.

In addition to translation regulation, inosine perturbs immunomodulatory RNA-sensing pathways through the destabilization of the secondary structures of Alu elements. The activity of inosine in retrotransposons possibly contributes to species evolution through their impact upon slicing sites, and the resulting generation of transcripts with alternative exon arrangements.

With the advances in RNA-seq and data processing, the landscape of inosine influence upon genomes, transcriptomes, and proteomes will become clearer and its impact upon human health will be better understood. Just as with so many other modified bases, we are just scratching the surface of inosine’s physiological significance.

## Data Availability

No new data were generated or analyzed in this study. Data sharing was not applicable.
